# Autophagy unrelated transcriptional mechanisms of hydroxychloroquine resistance revealed by integrated multi-omics of evolved cancer cells

**DOI:** 10.1080/15384101.2024.2402191

**Published:** 2024-09-19

**Authors:** Silvia G Vaena, Martin J Romeo, Mirna Mina-Abouda, Emma C Funk, George Fullbright, David T Long, Joe R. Delaney

**Affiliations:** a Hollings Cancer Center, Medical University of South Carolina, Charleston, SC, USA; b Department of Biochemistry and Molecular Biology, Medical University of South Carolina, Charleston, SC, USA

**Keywords:** Autophagy, hydroxychloroquine, colon cancer, ovarian cancer, drug resistance

## Abstract

Hydroxychloroquine (HCQ) and chloroquine are repurposed drugs known to disrupt autophagy, a molecular recycling pathway essential for tumor cell survival, chemotherapeutic resistance, and stemness. We pursued a multi-omic strategy in OVCAR3 ovarian cancer and CCL218 colorectal cancer cells. Two genome-scale screens were performed. In the forward genetic screen, cell populations were passaged for 15 drug pulse-chases with HCQ or vehicle control. Evolved cells were collected and processed for bulk RNA-seq, exome-seq, and single-cell RNA-seq (scRNA-seq). In the reverse genetic screen, a pooled CRISPR-Cas9 library was used in cells over three pulse-chases of HCQ or vehicle control treatments. HCQ evolved cells displayed remarkably few mutational differences, but substantial transcriptional differences. Transcriptomes revealed multiple pathways associated with resistance to HCQ, including upregulation of glycolysis, exocytosis, and chromosome condensation/segregation, or downregulation of translation and apoptosis. The Cas9 screen identified only one autophagy gene. Chromosome condensation and segregation were confirmed to be disrupted by HCQ in live cells and organelle-free *in vitro* extracts. Transcriptional plasticity was the primary mechanism by which cells evolved resistance to HCQ. Neither autophagy nor the lysosome were substantive hits. Our analysis may serve as a model for how to better position repurposed drugs in oncology.

## GRAPHICAL ABSTRACT

**Figure:**
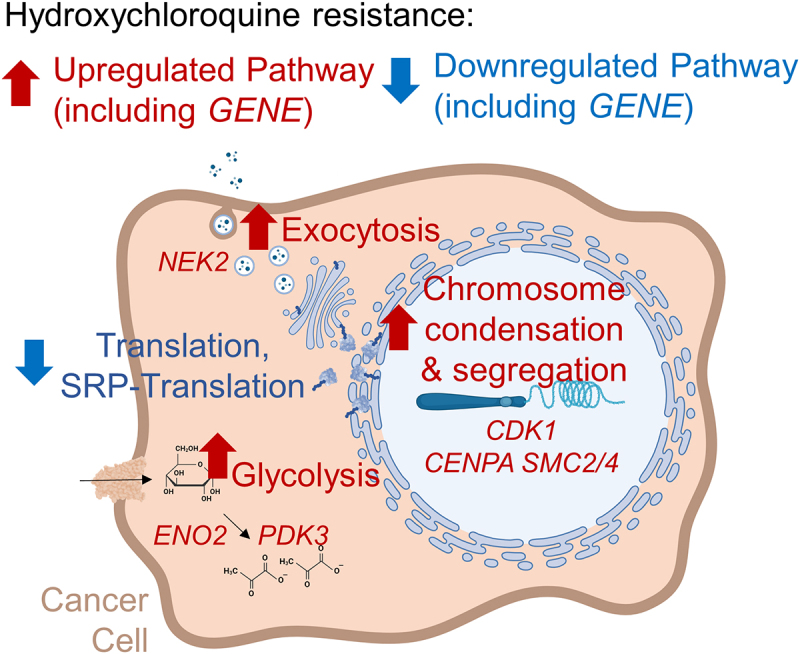

## Introduction

Chemotherapy resistance is a recurring problem in oncology, particularly in the era of precision medicine. Clinical trials often yield impressive progression-free survival benefits of oncoprotein-targeted therapies, only to fail to provide a lasting overall survival benefit. Mechanisms of resistance vary between cancer types as well as drugs administered. Precision medicines yield resistance mutations downstream in the molecular pathway or in a parallel molecular pathway to the targeted protein [1,2]. Broad-spectrum cytotoxic chemotherapeutics drive resistance mechanisms through transcriptional dysregulation or mutations of apoptotic programs, drug efflux pumps and/or cell stemness regulators, metabolism, or other more general cell biology programs [3,4]. While the hope is that oncologists will be able to target resistance mechanisms to precision medicine, many cancer types have yet to benefit from such strategies.

One strategy to best benefit cancer patients as rapidly as possible is to determine the mechanism of action of drugs which exhibit anti-cancer effects despite being originally intended for non-cancer indications [4]. The number of Food and Drug Administration (FDA) approved drugs now spans into the thousands. Many exhibit anti-proliferative effects on cancer cells. Drugs with existing approval require a New Drug Application (NDA) to the FDA to obtain a new cancer indication status, but most of the medicinal chemistry, pharmacodynamics, and safety data have already been performed during initial NDAs, associated clinical trials, and post-approval studies. With the expansion of next-generation sequencing (NGS) and other -omics strategies, there is an opportunity to study molecular pathways altered in cancer cells using promising repurposed drugs. Even those drugs originally developed for a single protein target often have useful off-target effects leading to cancer cell death [5]. Once these -omics studies are completed using drug repurposing candidates, better enrollment criteria may be designed for prospective oncology clinical trials. With this strategy to develop the molecular basis of action of a repurposed drug, a drug previously poorly or incompletely characterized in a cancer context can now be considered a precision medicine in the oncology space.

Hydroxychloroquine (HCQ), and its chemical cousin chloroquine, are antimalarial agents that impair hallmarks of cancer. The longest appreciated mechanism of action of HCQ on cancer cells is its ability to disrupt flux through the cellular recycling and metabolic pathway of autophagy [6,7]. Autophagy is essential in tumors for p53 suppression, adequate tricarboxylic acid cycle flux, nucleotide synthesis, angiogenesis, immune evasion, and survival during chemotherapy [8–12]. More recently, chloroquine was further shown to disrupt autophagosome-lysosome fusion concomitant with dispersion of canonical Golgi structures [13]. While other promising molecular agents targeting autophagy are under development, only 5% of cancer drugs pass Phase I studies [14] and there is evidence of osteoclast and neuronal toxicity risk in lysosomal-targeting strategies [15]. Conversely, HCQ or chloroquine have been shown to trend toward a benefit in many oncology clinical trials, including >12 months improved survival with chloroquine in glioblastoma multiforme [16,17], trends toward improved progression biomarkers in pancreatic cancer [18], a high objective response rate in colorectal cancer [19], with more limited responses in lung and breast cancers [20]. Our own research has implicated high-grade serous ovarian cancer as likely to benefit from HCQ due to multiple deletions of autophagy genes [21–23], of which *BECN1* is a haploinsufficient tumor suppressor of ovarian cancer [24]. Over 100 clinical trials in cancer have utilized HCQ or chloroquine, as listed on the clinicaltrials.gov database, indicating a need for improved biomarkers to guide patient selection and likelihood of benefit in such clinical trials.

To better understand how cancer cells evolve in response to HCQ-based therapy, we pursued a multi-omic strategy to define multiple mechanisms of HCQ response, genetic sensitization, and development of resistance. Using the chemoresistant high-grade serous ovarian cancer cell line OVCAR3 [25] and the chemoresistant female-derived colorectal adenocarcinoma cell line CCL218 [26], we performed forward and reverse genetic screens of modifiers of cancer cell viability following HCQ exposure. A comprehensive profiling of the genetic determinants of HCQ sensitivity and resistance are presented. This innovative systems biology approach enables an unbiased understanding of how pathways, rather than individual genes, allow for cancer evolution.

## Materials and methods

### Cells and culturing conditions

Complete RPMI consisted of 500 ml RPMI 1640 containing glutamine (VWR, #95042-508), 5 ml of sodium pyruvate solution (Sigma, #S8636-100 ML), 5 ml of penicillin streptomycin solution (Sigma, P4333-100 ML), and 50 ml fetal bovine serum (Thermo Fisher, #10437028). Cells were passaged within a day of surpassing 80% confluence by 1:10 dilution or according to cell density calculations denoted for each protocol. To remove cells from a plate, media were aspirated, cells washed once with sterile PBS (VWR, #45000-446), trypsinized with Trypsin-EDTA (Sigma, #T3924-100 ML) for 5–10 min, combined 1:1 with complete RPMI, and cell suspension centrifuged at 1,000 g for 2 min. Cells were either OVCAR3 (ATCC, NIH:OVCAR-3, #HTB-161) or CCL218, a colorectal cell line derived from HT29, validated by ATCC STR profiling.

### Spontaneous resistance forward genetic screen, library preparation, and deep sequencing

Cells were initially split from a 10 cm dish into a 96-well plate at a seeding density of 10,000 cells per well. For each subsequent passage, cells were briefly washed in 125 µL pre-warmed PBS and resuspended in 50–150 µL of pre-warmed 0.05% trypsin-EDTA, depending on each well’s cell density, with the higher volume corresponding to the highest cell density. Trypsinization lasted 10 min at 37°C. Cells were resuspended using a multi-channel P10 pipette set at 10 µL by vigorous pipetting 20 times for each well. Immediately following a row or column re-suspension, the multichannel pipette was used to transfer 10 µL of cells in trypsin to a new 96-well plate containing 140 µL normal media. Cells were allowed to adhere and proliferate for 24 h prior to drug addition. Drug was then added at 4X concentration (50 µL per well) and incubated with the cells for 48 h. Media were then aspirated using a P200 multichannel pipette and replaced with normal media for 2–10 days, depending on how long the cells required to recover (see Supplemental Figure S2). The concentration of drugs used for passaging the cells was 20–50 µM for hydroxychloroquine, reaching the 50 µM level at passage 7. At the final passage 15, plates were replicated to allow for RNA and DNA processing and scRNA-seq. Cells were grown without the presence of hydroxychloroquine for one week after the last drug exposure, to allow for examination of stably altered RNA independent of transient drug effects. Cells were pooled, representing one-third of each 96-well plate, pelleted, and processed by Genewiz for RNA-seq and whole-exome sequencing (WES). Qiagen AllPrep DNA/RNA kits were used to isolate RNA and DNA from the same sample. Agilent SureSelect Human All Exon V8 kits were used and polyA selection performed for transcriptomes. Next-generation sequencing (NGS) was performed on an Illumina HiSeq4000 using paired-end 150bp settings. For scRNA-seq, cells were processed similarly, but once pelleted, cells were resuspended in normal media and processed for 10X Chromium Controller genomics workflow (see scRNA-seq section).

### Bulk transcriptome analysis

Transcriptome FASTQ data was aligned to hg38 reference genome using STAR on the UseGalaxy platform [27]. RmDup removed duplicates. Transcripts were counted at the gene level using featureCounts, with a mean of 49.2 M gene counts per sample. Differential expression analysis used EdgeR using control-treated cells as controls. Pathway network analysis and network plots were performed using SWAN [23]. GO term enrichment analysis comparable to scRNA-seq analysis was performed using GOrilla [28], using ranked gene lists.

### Exome analysis

Exome FASTQ data was aligned to hg38 reference genome using Bowtie2. RmDup removed duplicates. UseGalaxy [27] was then used to create variant files and annotations. There was a mean coverage of 486X per sample. LoFreq was used with a minimal coverage of 10 reads and an FDR corrected *P*-value of ≥0.001. SnpEff and SnpSift were used to annotate VCF files, using database version GRCh38.86. VCF lines found in any control group or original cell line were removed from drug passaged VCF lines and considered background mutations. An allele fraction maximum of 0.8 was used to remove false positive calls unlikely to arise in a sub-pool of cells within the 96-well plate experimental setup. Only mutation calls with a “moderate” or “high” impact rating from SnpEff were used in the final analysis and reporting tables.

### Single-cell RNA-sequencing

Experimental procedures followed established techniques using the Chromium Single Cell 3’ Library v3.1 Kit (10× Genomics). Cultured cells (30,000 cells) in single-cell suspension were labeled with TotalSeq™-B anti-human Hashtag antibodies (Biolegend) and pooled in equal proportions prior to loading onto a 10X Genomics Next GEM Chip G and emulsified with 3’ Single Cell Next GEM beads using a Chromium™ Controller (10× Genomics). From barcoded cDNAs, gene expression libraries were constructed using Chromium™ Next GEM Single Cell 3ʹ Library kit and hashtag oligo libraries were constructed using Chromium™ Single Cell 3ʹ Feature Barcode Library Kit (both from 10X Genomics) at the Translational Science Laboratory (Medical University of South Carolina).

Next-generation sequencing was performed on each sample using an Illumina NovaSeq S4 flow cell at the VANTAGE facility (Vanderbilt University Medical Center). Partek Flow® was used to demultiplex samples, align reads to human genome hg38, flag and remove common contaminants including doublets, and generate RNA count tables per cell. ICARUS [29] was used to normalize data, remove high mitochondria read cells (>7.5% of reads), remove low expressing cells (<1,000 genes for CCL218 and <1,500 genes for OVCAR3), produce tSNE plots, Seurat clusters, cell cycle analysis and normalization. Average mapped gene reads for OVCAR3 were 24,787 per cell, with 831 cells in the control group and 754 in the HCQ resistant group. Average mapped gene reads for CCL218 were 20,230 per cell, with 704 cells in the control group and 4,008 in the HCQ resistant group. Differential expression analysis and SCENIC transcription regulon clustering was performed within ICARUS. GO term enrichment analysis of Seurat clusters was performed with GOrilla [28], using genes with *p* < 0.05 differential expression and >0.1 magnitude change and hg38 genes as a background list. Copy-number alteration analysis utilized InferCNV (inferCNV of the Trinity CTAT Project, https://github.com/broadinstitute/inferCNV, using an i3 hidden Markov model transition probability of 1 in 1,000, a window length of 300, and a cutoff of 0.1.

### CRISPR-Cas9 screen biological steps

The Brunello human CRISPR-spCas9 (Cas9) 1-vector pooled sgRNA library was used, which contains 76,441 sgRNAs for 19,114 human genes and 1,000 non-targeting guides and spCas9 on the same plasmid with the lentiCRISPRv2 backbone. Human Brunello CRISPR knockout pooled library was a gift from David Root and John Doench (Addgene #73179-LV). Viable cells were counted using a Biorad TC20 and seeded at 3.1 million viable cells per 10 cm tissue culture plates (VWR, #10062-880), with 5 plates per replicate and two replicates per condition. Cells were seeded in complete RPMI in the presence of 0.1% polybrene and infected with the CRISPR-Cas9 Brunello library at a multiplicity of infection of 0.5 active viral particle units per viable cell. Twenty-four hours after transduction, the media were replaced with complete RPMI containing 2 µg/ml puromycin. Media contained puromycin from this point onward for the remainder of the screen. Seventy-two hours post-transduction, each stack of five 10 cm tissue culture plates was passaged using Trypsin-EDTA and an aliquot of 15.5 million dry pelleted cells collected for genomic DNA isolation for NGS of sgRNAs encoded on the transduced plasmids. This results in ~100 cells genotyped per each individual sgRNA. Another 15.5 million cells were evenly plated onto five 10 cm tissue culture plates, resulting in 3.1 million cells per plate in parallel. Drug was added during cell re-plating, using either 20–50 µM hydroxychloroquine (TCI America, #H13065G) or an equivalent amount of DMSO diluent (0.0125%, Sigma, D8418-100 ML) to vehicle control cells. Since this protocol resulted in a 50% growth inhibition rate over 4 days in quality control experiments, vehicle control cells were re-seeded at 8.37 million viable cells per set of five 10 cm plates, resulting in a manual 50% depletion of cells. Forty-eight hours after re-plating with drug, media are replaced with drug-free complete RPMI. Forty-eight hours after fresh media addition, control cells are always collected for another round of plating and genomic DNA isolation for NGS. HCQ treated cells were allowed additional days to grow until plates were as confluent as the control cells, as needed. In total, four cell pellets were collected per whole-genome screen: (1) starting cells, (2) single-treatment cells, (3) cells which grew after two drug treatments, and (4) cells which grew after three drug treatments.

### CRISPR-Cas9 screen NGS library construction, sequencing, and bioinformatic analysis

Genomic DNA was first isolated using four micro columns per cell pellet (Invitrogen, #K182002) according to manufacturer instructions. Each column was eluted twice with 40 µl elution buffer and elutions from the same cell pellet pooled prior to NGS library preparation. PCR and NGS library construction using Illumina-based TruSeq primers were performed according to Brunello library supplier protocol (Addgene), using an equal amount (5-7µg) of genomic DNA starting material for each whole-genome screen isolate. Libraries were pooled and sequenced on an Illumina NovaSeq-6000. FASTQ files were demultiplexed and count tables generated using perfect sequence matching in R (code available at https://github.com/jrdelaney/Published_Code/ file sgRNA_counter_HCQevolPub.R), with sgRNAs required to follow directly after NGS primers. The average count per replicate per sgRNA was 44.9, and the average count per replicate per gene was 180 (x2 replicates, x4 time points). To determine significant hits, MAGeCK was used [30]. The MLE module was used to create P-values utilizing the time-series performed in this experiment. Nominal drug Wald-*P*-values of *p* ≤ 0.05 were used as the primary significance criteria in each cell line for display and comparison purposes. Complete MAGeCK statistics per gene are available in Supplemental Table S5.

### Cell viability assays

To test cell pool viability, cells were identically passaged as in the spontaneous resistance section, but transferred to separate plates: (a) for control cell growth and (b) for drug treatment. Each plate contained three blank wells and the minimum staining value was used as a blank for each plate. Crystal violet staining was performed as previously described [21,22] after 48 h. The drug treated plate was compared to the control treated plate to form a cell loss ratio = (1-drugA600/controlA600)*100 for each individual well. Wilcoxon rank sum statistical comparisons utilized each well containing cells (*N* = 93) per plate, ranked from the most resistant well to the most sensitive well. Wells with staining artifacts, defined as more than 100% cell loss or negative growth inhibition, were removed from the analysis. Cells passaged in normal media were used to determine the nonresistant null distribution.

### H2B-BFP live cell microscopy

CAOV3 cells were transfected with BFP-H2B (Addgene #55243, EBFP2-H2B-6 was a gift from Michael Davidson) and seeded at a density of 2,500 cells per well. Cells were treated with 33 µM HCQ or DMSO vehicle control and imaged live on an Agilent BioTek Lionheart FX microscope. Imaging started immediately post treatment, capturing an image every 10 min for 72 h. Acquired images were then removed of labels to enable blinded quantitation of cell division processes. The data were manually quantified by the blinded observer by counting the number of tripolar divisions in HCQ treated and control cells. For chromatin compaction and decompaction pre- and post-mitosis, respectively, the blinded observer noted the number of frames from metaphase for both processes for each metaphase assessed. Data from 957 metaphases were tabulated, with at least 100 metaphases per condition, from two independent experiments. Tabulated results were then unblinded for statistical analysis and graph preparation. For interphase analysis, the number of frames between total decompaction and compaction was quantified for 30 cells each in HCQ and control treatment conditions.

### Reactions in xenopus egg extract

*Xenopus laevis* were cared for and used according to approved IACUC and AAALAC protocols. *Xenopus* egg extracts and sperm chromatin were prepared as previously described [31]. Briefly, nucleoplasmic extract (NPE) was formed by incubating demembranated sperm chromatin in a crude egg lysate to form nuclei. The nuclei were then isolated by centrifugation and fractionated by ultracentrifugation to remove lipids and chromatin. For reactions, NPE was supplemented with 1 mM DTT and ATP regenerating mix (6.5 mM phosphocreatine, 0.65 mM ATP, and 1.6 µg/mL creatine phosphokinase) and incubated with 1,250 demembranated sperm chromatin units per µL. Where indicated, reactions were also supplemented with 500 µM hydroxychloroquine. At the indicated time points, samples were withdrawn and visualized by phase contrast light microscopy.

### Statistical analysis

Specialized algorithms reported *P*-values specific to the tool used for each next-generation sequencing project. A fisher’s exact test was used to calculate statistics for 2 × 2 contingency tables, notably the tripolar division counts compared to normal metaphase counts in BFP-H2B live cell microscopy. For viability assays, a Wilcoxon rank sum test between colony tests was used to generate *P*-values. For all other assays, an unpaired two-sided student’s t-test was performed.

## Results

### Forward genetics HCQ-resistant screen design and initial quality control

**Figure 1.**
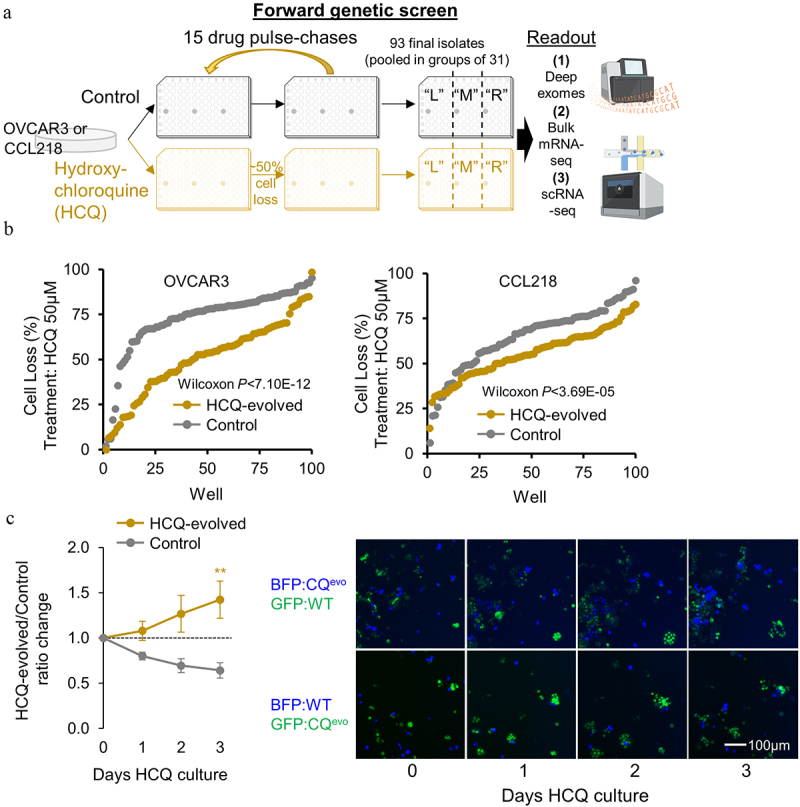
Design of multiplexed stochastic HCQ-evolution study and quality control. (a) schematic of experimental design to evolve and measure mechanisms of HCQ resistance in 93 independent evolved or control cultures. (b) quality control viability assay results on cells after 6 pulse-chases of HCQ, ordered by rank per well of HCQ sensitivity. (c) adapted CCL218 pools were labeled lentivirally with GFP or BFP after 15 passages and co-cultured in the presence of HCQ. Quantitation of HCQ-evolved cells and control cells relative to starting ratios are shown. ** indicates *P*<0.01 by t-test.

To investigate how cancer cells may evolve independent mechanisms of drug resistance, we performed a multi-omic forward genetic screen on individually passaged small pools of cells. 96-well plates were seeded with equal numbers of cells per well with three empty wells for quality control. One plate was passaged under control conditions, and a paired plate was treated with a 48 h pulse of HCQ, killing approximately 50% of cells, and allowed to recover in normal media. Sequential pulses were performed until HCQ treated cells received 15 drug exposures (Figure 1(a)). Each well was always seeded into the same well for each passage, allowing for 93 independent experiments to evolve drug resistance. Ovarian cancer OVCAR3 cells and colorectal cancer CCL218 cells, which are p53 mutant, platinum-resistant, and high in copy-number alterations, were used as cell line models. An interim analysis demonstrated HCQ-evolved plate was less sensitive to cell loss by HCQ exposure than the control plate (Figure 1(b)) and centered near 50% cell loss.

At the final passage, plates were duplicated and cells pooled in groups of 31 well isolates according to plate position (Figure 1(a)). Each individual pool was processed for (1) deep exome sequencing at >400X coverage (486X average), (2) bulk mRNA-seq, and all three cell pools were combined for (3) scRNA-seq. As further validation of stably resistant cells, pools were labeled with either GFP or BFP and placed in a competition experiment. HCQ-evolved cells outcompeted the control cells during exposure to HCQ (Figure 1(c)).

### Exome mutations discovered in forward genetic screen of HCQ-resistant cells

In total, using low-frequency variant calling (see Methods), 114 unique SNVs were found in OVCAR3 cells and 84 unique SNVs were found in CCL218 cells (Supplementary Table S1). Interestingly, none were marked as known oncogenic in the COSMIC database of cancer mutations [32], suggesting these SNVs may be novel modulators of tumor biology. Between the two cell lines, 8 SNVs were shared. Of these, there are two likely false positives: CR1L-I455V, which has no RNA expression in resistant cells, and RGPD3-H1284R which is within a highly duplicated family of RANBP2 genes and may have alignment artifacts. ZNF638 and FCGBP have mutations with a high allelic fraction within their pool (>0.6), indicating a strong presence in all HCQ-resistant wells within their pool, which is unlikely spontaneous and are probably artifacts of unknown origin. The remaining four are most likely to be attributed to HCQ resistance, as they were found at reasonably low allelic fractions and present in RNA expression. SMOX, with a C448Y mutation, is a flavoenzyme which oxidizes spermine to create spermidine. Spermidine is a known inducer of autophagy [33], histone deacetylation, and regulator of reactive oxygen species [34]. A mucosal protein hit was found: MUC3A, with a E725K mutation. TREML2-D23E was mutated in both cell lines. TREML2 is a cell surface receptor with controversial interaction with CD276 involved in the innate and adaptive immune response [35]. STAG2, with a A710S mutation, is a component of the cohesin complex and is required for efficient chromosome segregation. Within cell lines, mutations were most often associated with a single pool of cells, consistent with spontaneous generation. Some mutations were present in low allele fractions yet were nonetheless identified as mutant in multiple pools of cells. Notably, none of the HCQ-resistance mutations described here were found in control treated cells or the original starting cell culture, as these were removed during bioinformatic analysis. CTR9-A655D was found in all OVCAR3 HCQ-resistant pools at low allelic fraction, and its mRNA is expressed in the resistant pools. CTR9 is a component of the PAF1 complex involved in mRNA quality control and transcriptional elongation [36], and CTR9 mutations are linked to tumorigenesis [37]. CCL218 exhibited more hits with similarly low allelic fraction yet were present in all CCL218 pools. OR52I2-T167M, DMWD-G88R, AP2A1-T242P, DNAH11-A3527D, and MOSPD1-G166E SNVs were in all HCQ-resistant pools at an allelic fraction less than 0.4. Taken together, no single driver mutation was involved in HCQ resistance, although genes involved in redox homeostasis and chromosome segregation were found.

### Transcriptome differences of HCQ-resistant cells

**Figure 2.**
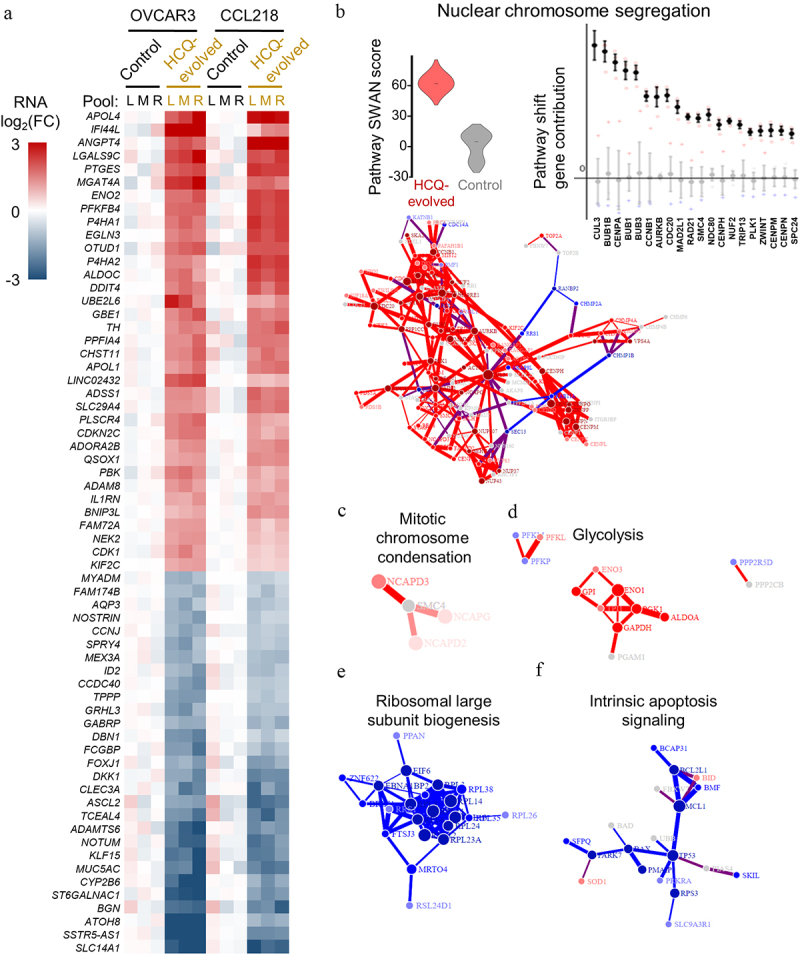
Bulk transcriptomic analysis of HCQ evolved cell pools. (a) significant (FDR < 0.05) differentially expressed genes associated with HCQ-resistance in both cell lines. (b-f) SWAN pathway network analysis of HCQ-evolved transcriptomes of labeled pathways. Blue represents suppressed interactions, red represents elevated interactions.

RNA was collected at the end of the screen, after a one-week drug washout period. Bulk transcriptome analysis was performed on each resistant pool compared to control. HCQ resistant OVCAR3 cells yielded 63 genes which were significantly and consistently overexpressed relative to controls, of which 35 were also significantly and consistently upregulated in CCL218 cells. HCQ resistant OVCAR3 cells were suppressed in 115 genes, of which 29 were shared with CCL218. HCQ-resistance associated gene sets are presented in a heat map in Figure 2(a). The most overexpressed gene *APOL4* is a primate-specific apolipoprotein associated with endothelial cell lipid metabolism [38] (Supplemental Table S2). The most suppressed gene *SLC14A1* encodes a urea transporter with similarity to ion channels [39], perhaps related to HCQ disruption of osmolarity [40]. SWAN pathway network analysis revealed that one of the most upregulated pathways in both cell lines was Nuclear Chromosome Segregation (Figure 2(b), Supplemental Table S3). Dysregulated chromosome segregation was from elevated networks including *CUL3*, *BUB* genes, and centromeric core histone genes. Mitotic Chromosome Condensation was also upregulated in both sets of resistant cells, largely through upregulation of Condensin complex genes *NCAPG*, *NCAPD2*, and *NCAPD3*, which interact with SMC1A/2/4 to condense chromatin [41] (Figure 2(c)). Metabolic dysregulation was also observed. Nucleotide metabolism was altered, including ATP, ADP, and NAD metabolism (Supplemental Table S3). The top hit shared pathway was glycolysis, led by enolase genes, including ENO1 which converts 2-phosphoglycerate to phosphoenolpyruvate and is also involved in hypoxia response (Figure 2(d)). Upregulated pathways thus centered on metabolism and chromosome condensation and segregation.

Transcriptional suppression included Ribosomal Large Subunit Biogenesis (Figure 2(e)). While this may be predicted to coordinately elevate stress response pathways, stress response upregulation was not shared between resistant cell line pools. One exception was upregulation of hypoxia gene networks was observed in HCQ resistant cells (Supplemental Table S3). A suppression of Intrinsic Apoptosis Signaling gene networks was also observed (Figure 2(f)), but this is unlikely to be specific to HCQ. Suppressed transcriptional programs were thus general and not HCQ specific.

### Single-cell RNA sequencing of evolved cells uncovers variable resistance programs

**Figure 3.**
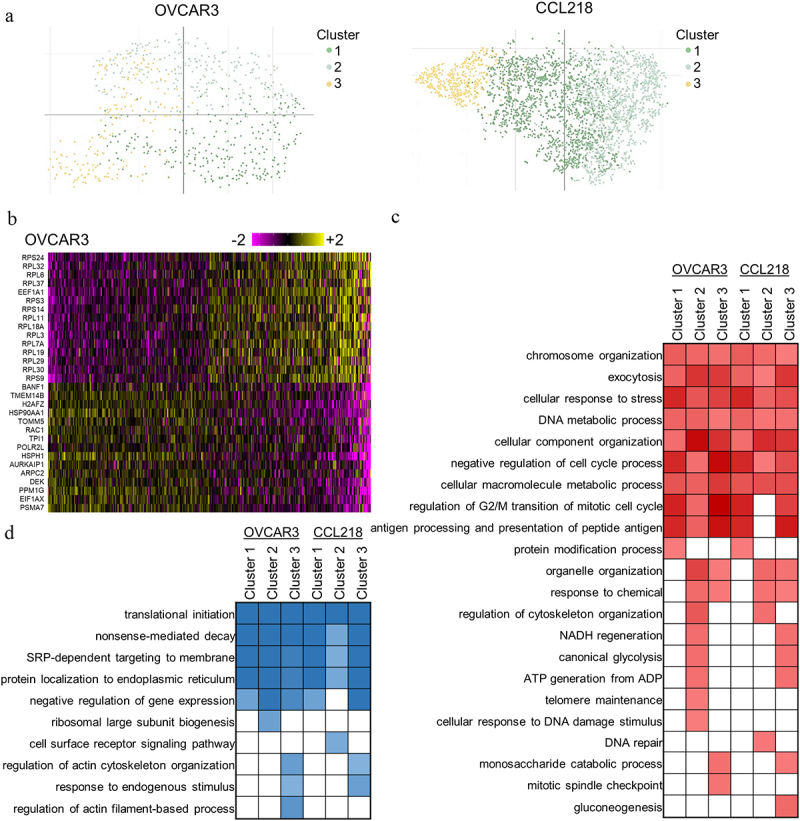
Single-cell RNA-seq of HCQ-evolved cells. (a) tSNE plots of HCQ-evolved cells, corrected for cell cycle. Seurat clusters represent the yellow, light green, and dark green coloring. (b) differentially expressed genes within the scRNA-seq clusters of OVCAR3. (c) comparison of altered pathways to Seurat clusters in the scRNA-seq dataset. Coloring is proportional to upregulated pathway –log10 significance (red) or (d) downregulated pathway significance values (blue). White fill represents pathways that were not significantly altered.

While bulk RNA sequencing can have greater statistical power and reliability across more expressed transcripts, single-cell RNA sequencing (scRNA-seq) enables a deeper investigation of heterogeneity. A recent pan-cancer analysis of scRNA-seq data found independent meta-programs within and between cancer types, inferred as evidence of selective pressure [42]. Here, the selective pressure applied is strictly due to drug response. Cells processed in parallel to those used for bulk sequencing were subjected to scRNA-seq library preparation and sequencing. Three Seurat clusters for each cell line were identified (Figure 3(a)), which are notably a gradient rather than distinctly separated. A prominent distinction in the OVCAR3 HCQ-evolved pool was a differential downregulation of the large ribosomal subunit genes (Figure 3(b)) which was separate from cells upregulating a mixture of other resistance pathways. These Seurat-defined cell clustered RNA patterns were next compared to each other and those found by bulk RNA-seq.

Top pathways with significant downregulation or upregulation observed in the bulk RNA analysis were again significantly altered in this scRNA-seq dataset (Supplemental Table S4). Upregulated pathways in all cell clusters included chromosome organization, exocytosis, response to stress, DNA metabolism, cellular component organization, negative regulation of the cell cycle, regulation of the G2/M transition, and antigen processing and presentation of peptide antigens. Downregulated pathways observed in all clusters include translational initiation, nonsense-mediated decay, protein localization to the endoplasmic reticulum (ER), and signal recognition particle dependent co-translational protein targeting to the membrane.

Pathway alterations specific to sub-populations of cells, that is, clusters, were also observed. Alternate resistance pathways were present in the cell pools. Cluster 1 was instead associated with upregulation of protein modifications and devoid of most other cluster-specific dysregulation. Clusters 2 and 3 upregulated organelle organization, response to chemical, ATP generation from ADP, and glycolysis. Cluster 2 was unique in its upregulation of telomere maintenance, response to DNA damage stimulus, and DNA repair, as well as uniquely downregulating the cell surface receptor signaling pathway. Cluster 3 uniquely downregulated the response to endogenous stimulus and the regulation of the actin cytoskeleton, while uniquely upregulating the mitotic spindle checkpoint (OVCAR3) or, strangely in opposition to its upregulated glycolysis, the gluconeogenesis pathway (CCL218). Stress response pathways were associated with the ATF4 transcription factor, a canonical activator of ER stress [43], in regulon analysis (Supplementary Figure S1). Cohesin complex assembly was associated with RAD21 and SMC3 regulons, whereas cell cycle regulation was related to MYC and ETS2. The CEBPB regulon was identified as involved in dysregulation, although it likely has varying roles in glucose metabolism, exocytosis, and antigen presentation.

Altogether, a majority of the altered pathways discovered in the bulk RNA-seq study were dysregulated in a smaller subset of clusters (Supplementary Table S3). These results indicate that multiple independent transcriptional programs were associated with resistance to hydroxychloroquine.

### Contribution of copy-number alterations to cellular evolution

**Figure 4.**
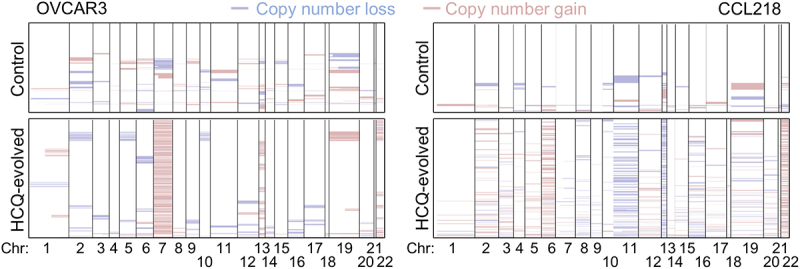
Single cell copy-number alterations of resistant cells. InferCNV copy-number alterations relative to unaltered control cells. Each horizontal row represents a single cell.

Copy-number alterations are well known causes of transcriptional and protein-level changes in cancer cells [44]. To investigate if the observed altered pathways had a copy-number alteration component, InferCNV was utilized to discover chromosome-arm level genetic aberrations resulting in extra gene copies or loss of gene copies in the scRNA-seq cells. Stochastic alterations were observed in control cells and HCQ-adapted cells (Figure 4, top panels), but evidence of chromosome selection was also observed in HCQ-adapted cells (Figure 4, lower panels). Chromosome 22 was positively selected in both OVCAR3 and CCL218 populations (Fisher’s exact test *p* < 0.0001). Chromosome 7 (*p* < 0.0001) and 13 (*p* < 0.0001) were positively selected in OVCAR3 cells, while losses were not enriched in the resistant cells. Chromosome 6 was positively selected in CCL218, while chromosomes 11 and 13 were selected for losses in HCQ-resistant cells (*p* < 0.0001). While dozens to hundreds of genes are present on each of these chromosomes, some of the most upregulated transcriptional pathways have genes encoded on the gained chromosomes. Actin nucleation relies on *ARPC1A/B* and *ACTR3B*, which are encoded on Chromosome 7. Cytochrome C, a major component of both the electron transport chain and a signaling molecule for intrinsic apoptosis, is also encoded by *CYCS* on chromosome 7. Chromosome 22 encodes two primary unfolded protein response regulators: *XBP1* and *ATF4*.

### CRISPR-Cas9 orthogonal validation screen of HCQ sensitivity modulators

**Figure 5.**
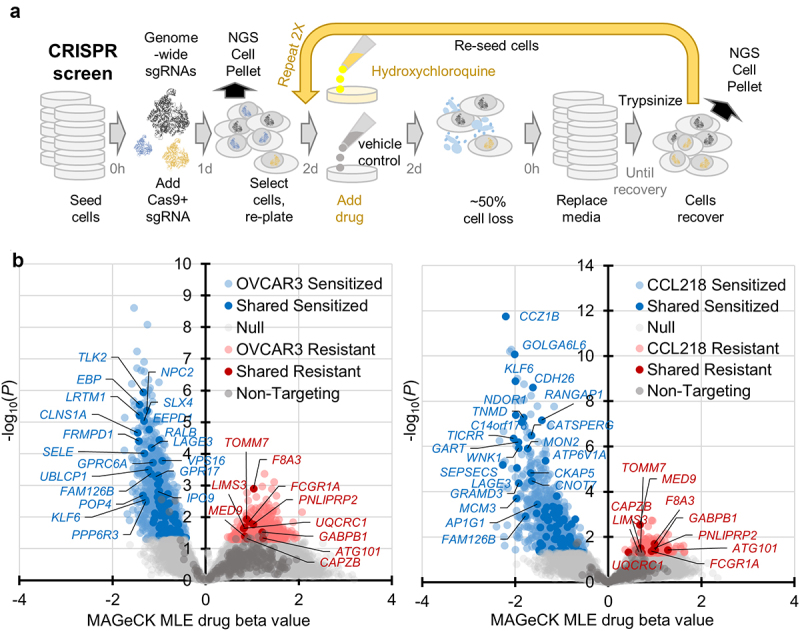
CRISPR-Cas9 screen of functional HCQ viability modulators. (a) schematic of genome-scale CRISPR-Cas9 screen protocol. (b) volcano plots of hit genes, with top genes found as hits in both cell lines (“shared” sensitized or resistant) labeled. Knockouts which survived better than controls over time in the presence of HCQ are displayed in red, those knockouts which sensitized cells to more death in the presence of HCQ are shown in blue. Beta values represent the magnitude of altered viability.

While the transcriptional changes observed were the most striking of all changes measured, causal roles are difficult to infer. Reverse genetic screens directly implicate genes with their ability to confer enhanced survival or cell death sensitivity. We opted to perform a genome-scale screen of HCQ sensitivity modulating genes through the Brunello CRISPR-Cas9 genome-scale library targeting 19,114 genes. Two controls were used in the analysis: cells passaged under vehicle control conditions and 1,000 non-targeting guides present in all cell pools. The experimental condition was HCQ treatment with a target 50% growth inhibition (see Methods). Higher than expected cell loss was observed during the screen despite quality control tests preceding the assay mimicking all conditions (Supplementary figure S2), indicating there may have been an interaction between DNA breaks caused by Cas9 and HCQ treatment. Nonetheless, pools of cells within all replicates survived and proliferated throughout the screen, enabling a time-course analysis of three drug exposures followed by recovery (Figure 5(a)). Cells were isolated following each recovery period and subjected to NGS procedures allowing for sgRNA quantitation.

A time-course analysis using the MAGeCK MLE module [30] enabled a quantitative assessment of knockout genes which conferred either sensitivity to HCQ or resistance to HCQ. Altogether, 1,183 knockout genes nominally (drug Wald-*P*-value) sensitized OVCAR3 cells and 788 knockout genes sensitized CCL218 cells, with 82 genes in common (Figure 5b, supplementary Table S5). In OVCAR3, 561 gene knockouts conferred resistance to HCQ and 227 knockouts in CCL218, with 10 genes in common. There was no pathway enrichment in either common gene set (all FDR > 0.05, by GOrilla [28]). There was only one autophagy gene hit, in the resistance-conferring knockout group, *ATG101*. ATG101 may be a unique target as it is not homologous to other autophagy proteins [45]. ATG101 is essential for autophagy, stabilizing ATG13 in the ULK1 autophagy initiation complex and connecting to phosphatidylinositol 3-kinase complexes containing PIK3C3 and BECN1 [46]. As HCQ disrupts the Golgi, Golgi-related hits were also of interest. Again, there was only one directly related hit, *GOLGA6L6*, which sensitized both cell lines to HCQ upon deletion. However, it is thought to be a pseudogene, and was indeed not expressed in either cell line in our analysis. HCQ affects lysosomal acidity. Of all V-ATPase components within our screen, the *ATP6V1A* gene was the top hit, sensitizing both cell lines significantly to HCQ. Many other dual cell line hits were sensible in context with the transcriptional resistance programs associated with resistance (Figure 2).

### Integrated analysis of HCQ resistance

While each assay presented herein specializes in certain types of HCQ adaptation, results synergize when compared to enable an understanding of the most biologically relevant individual genes. One hypothesis is that upregulated genes within the RNA-seq adaptation experiment are upregulated in response to adaptive programs (such as the regulons in Supplementary Figure S1), yet only a subset of these upregulated genes directly confer drug resistance. Similarly, hit genes within the CRISPR-Cas9 study may generally be involved with a variety of drugs’ sensitivity modulation, and only a subset is most directly related to HCQ. However, hit genes within multiple assays can dramatically refine which are most directly involved in HCQ viability modulation. Only a small handful were both transcriptionally upregulated and also hit sensitizers in the same cell line by CRISPR-Cas9 screening (summarized in supplementary Table S6).

**Figure 6.**
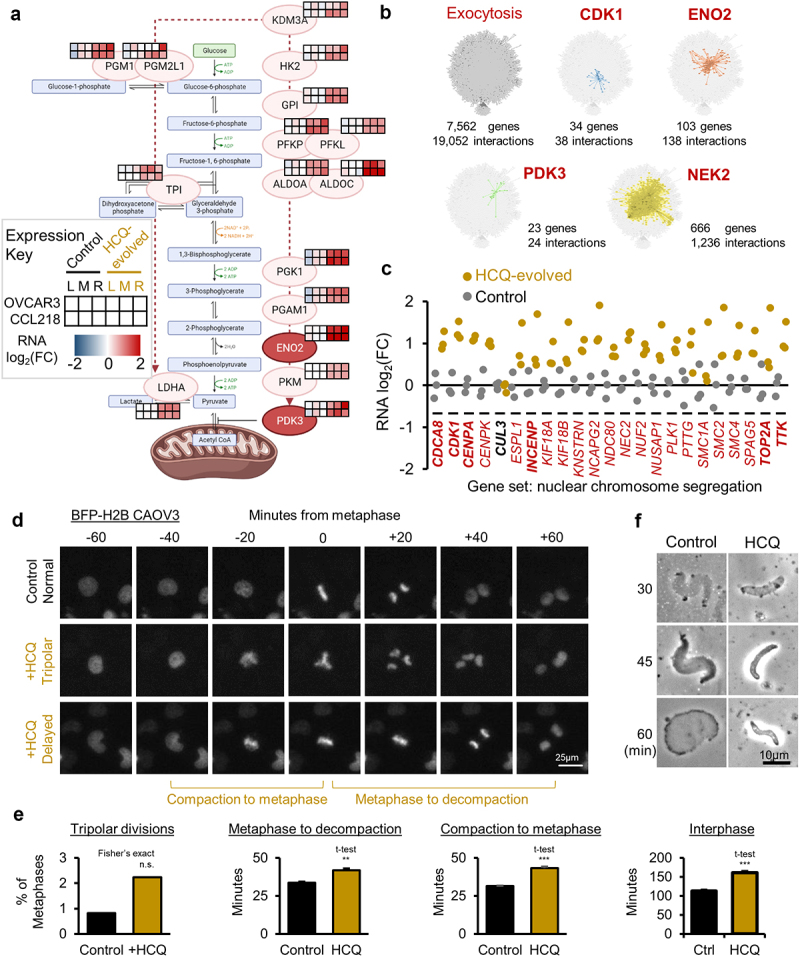
Integrated analysis and validation of HCQ resistance modulation. (a) aerobic glycolysis upregulated genes (light red) in HCQ-evolved cells, with CRISPR-Cas9 sensitizers additionally highlighted (dark red), relative to their action points along the glycolytic pathway (light blue). Expression heat maps are shown next to each gene, with red representing upregulation magnitude in the three hcq-evolved transcriptome pools. Heat maps show OVCAR3 cells in the first row, CCL218 cells in second row, control samples in the first three columns, and evolved samples in the last three columns. (b) the exocytosis protein–protein interaction network (upper left) is split into subnetworks connected to indicated transcriptional and CRISPR screen dual hit genes (colored panels). Interactions are from human BioGRID, with network visualization from Cytoscape. (c) transcriptionally upregulated genes (red) in HCQ evolved cells or CRISPR-Cas9 hcq-sensitizing knockouts (bold) within the nuclear chromosome segregation pathway. (d) CAOV3 cells transfected with BFP-H2B and observed by live-cell microscopy at 10 min intervals. Top row shows a normal division in vehicle treated controls. Middle row shows an abnormal tripolar division in HCQ treated cells. Lower row shows abnormally slow chromatin compaction prior to metaphase formation and slow segregation/de-compaction following metaphase in HCQ treated cells. (e) blinded quantitation of events shown in D from *N*=2 independent experiments.(f) sperm chromatin was incubated in NPE at 21°C for 15 min. Reactions were split and supplemented with vehicle or HCQ. At the indicated time points, samples were withdrawn and visualized by phase contrast light microscopy. Representative images from *N*=3 experiments.

Aerobic glycolysis is often a feature of cancer metabolism, and we found it was transcriptionally upregulated, more so in one cell cluster from each cell line. Chloroquine has previously been shown to inhibit glycolysis and impair mitochondrial function [47–49]. Two CRISPR screen hits fulfill the thesis that HCQ-resistance requires aerobic glycolysis: *ENO2* and *PDK3* knockouts sensitized cells to HCQ (Figure 6(a)). ENO2 is enolase 2, which converts 2-phospho-glycerate to 2-phosphoenolpyruvate in glycolysis. Overexpression of ENO2 is sufficient to increase glycolysis in cancer cells [50]. PDK3 is a HIF-1 induced pyruvate dehydrogenase kinase [51], which inhibits pyruvate dehydrogenase conversion of pyruvate to acetyl-CoA. KDM3A, an upregulated gene, is an oxygen-sensing lysine demethylase which coordinates mitochondria biogenesis through PGC1α [52]. *KDM3A* overexpression is unique among the KDM3 subfamily in cancer, conferring colony formation, migration, invasion, and metastasis through Hippo and YAP1 pathway upregulation [53] Glycolytic genes and glucose transport genes, such as *GLUT3/SLCA3*, are directly activated by KDM3A and HIF1 [54]. KDM3A acts in a feed-forward loop with HIF1 to upregulate glycolytic genes [55]. All upregulated glycolytic genes found in HCQ resistant cells are graphed in Figure 6a.

Of all transcriptionally upregulated genes, *NEK2* was most involved in the exocytosis network. *NEK2* was also a CRISPR-Cas9 screen sensitizing hit. The exocytosis pathway, a transcriptionally upregulated program in HCQ resistance, may most directly connect to known molecular biology effects of HCQ: inhibition of autophagosome-lysosome fusion and disruption of the Golgi [13]. Proteins bound for exocytosis must travel through the Golgi but may do so in a lysosome dependent or independent manner [56]. Cancer cells may upregulate exocytosis due to the deleterious protein trafficking effects induced by HCQ. The exocytosis GO pathway consists of 236 highly interactive proteins [57], resulting in an interaction network involving 7,562 proteins and 19,052 interactions, as annotated in BioGRID [58]. Using Cytoscape [59], subnetworks centered on RNA-seq upregulated genes within the exocytosis network were drawn and quantified (Figure 6(b)). NEK2 is a known regulator of kinetochore-microtubule attachment and timing [60,61], centrosome stability [62], and cell stemness, including drug efflux pumps [63,64], all of which were shown here to be associated with HCQ resistance.

Mitotic chromatin condensation and kinetochore attachment programs are clearly critical for HCQ resistance. Of the upregulated genes, *CENPA* and *CDK1* were also sensitized hits within the CRISPR-Cas9 screen (Figure 6(c)). Mutations in *STAG2*, a component of the cohesin complex, also altered this pathway in resistant cells. While the cullin-RING E3 ubiquitin ligase CUL3 itself was not upregulated in resistant cells, its interaction network was (Figure 2(b)), and *CUL3* was a significant CRISPR-Cas9 screen sensitizing hit in CCL218. Protein quality control is essential for appropriate mitotic protein network function. The observed reduction of translation-related mRNAs may further contribute to mitotic fidelity by slowing protein production and reducing the burden on quality control mechanisms. To ascertain if HCQ directly impairs chromatin compaction and decompaction, important aspects of accurate chromosome segregation during mitosis [41], we assessed chromatin dynamics by live-cell fluorescent microscopy. BFP-H2B was used to label chromatin in CAOV3 ovarian cancer cells. Cells were examined after addition of HCQ (33 µM) or vehicle control. Three classes of chromatin condensation and segregation were assessed: tripolar divisions, compaction time to metaphase, and decompaction time from metaphase (Figure 6 6(d)). Images were blinded and results tabulated from blinded data. Following HCQ treatment, there was a trend toward an increase in abnormal tripolar divisions (*p* < 0.16) and a significant delay in chromatin compaction or segregation and decompaction before and after metaphase, respectively (Figure 6(e)). Interphase was also significantly slowed, as expected from HCQ’s role in metabolic and autophagic dysregulation. Nuclear extracts do not contain lipid-based organelles such as lysosomes nor autophagosomes [65], allowing for autophagy-independent biochemical analysis. We additionally found HCQ to slow chromatin decompaction in these nuclear extracts (Figure 6(f)). Thus, this HCQ-induced chromatin dysregulation phenotype may drive the transcriptional program upregulation of chromosome segregation and organization observed in HCQ-resistant cells.

## Discussion

Here, we describe a bulk transcriptomic, single-cell transcriptomic, exomic, and CRISPR-Cas9 genome-scale analysis of how cancer cells can acquire resistance, using HCQ as an example of a repurposed drug. Like many repurposed drugs, HCQ does not impinge a single target protein, but rather has been observed to disrupt entire molecular pathways, which we also observed here. We discovered that an increase in glycolytic enzymes, an increase in exocytosis regulators, an increase in mitotic control of chromosome condensation and segregation, and a decrease in translation all contribute to mechanisms of resistance to HCQ.

Our results support a model for transcriptional plasticity as a primary mechanism of drug resistance, as opposed to SNV/indel mutations and copy-number alterations. Recent research utilizing whole-genome sequenced samples to track evolutionary divergence came to the conclusion that clones that give rise to metastasis and recurrence are often already present early in cancer development [66]. This is consistent with findings that copy-number alterations are relatively stable during disease progression [67] and less correlated to protein content in a chemo-refractory state [68]. In our well-controlled *in vitro* conditions, cells never created the same gene deletions that were found in CRISPR-Cas9 mediated deletions which induce HCQ resistance. Gene deletions of such targets may be considered the “easiest” path to drug resistance in a genome unstable cell. However, transcriptionally upregulated pathways and genes were found to overlap those Cas9-induced gene deletions known to confer sensitization. Single cancer cells acquire divergent transcriptional paths to resistance. Yet, pools of tumor cells also acquire convergent transcription pathway suppressions and elevations. Thus, while cancer cells are indisputably higher in mutation rate than normal cells, their ability to modulate transcriptional plasticity is the primary mode of therapy resistance found here.

Dysregulation associated with HCQ resistance was consistently affects processes involved in proper chromosome segregation and chromatin regulation. Our direct evidence demonstrates that chromosome segregation is aberrant upon HCQ exposure. Specifically, HCQ exposure slows chromatin compaction and decompaction processes, particularly during mitosis. Cells that have evolved to survive HCQ outcompete control cells, exhibiting a faster cell cycle in the presence of HCQ (Figure 1(c)). These findings support the hypothesis that cancer cells upregulate transcriptional programs related to chromosome segregation in response to HCQ-induced stress. However, our results are correlative, and it remains uncertain whether genetic experiments designed to upregulate chromosome segregation signaling will confer HCQ resistance. Our CRISPR-Cas9 screen results suggest that knocking out chromosome condensation and segregation core regulators results in sensitization to HCQ (Figure 6(c)). Future genetic studies investigating our multi-screen hits will enhance our understanding of how autophagy and HCQ interact with specific programs regulating cancer cell viability and resistance.

HCQ or chloroquine phosphate have been used in hundreds of studies to prohibit proper autophagic flux. Given this well-known function of HCQ, it was expected that canonical autophagy genes would be positive control hits regulating sensitivity or resistance to HCQ. Contrary to this hypothesis, mutated or dysregulated canonical autophagy initiation genes were not found in any resistant cell line pool, and only one, *ATG101*, in both CRISPR-Cas9 screens. This might be expected in hindsight for two reasons. First, HCQ is known to dysregulate Golgi structure and trafficking which are necessary for exocytosis [13]. SNARE complex genes essential for autophagosome-lysosome fusion, which are disrupted by chloroquine, *STX17* and *SNAP29* [13,69], surprisingly were not hit on any screen herein. Second, we previously found that rapamycin, an mTORC1 inhibitor and inducer of autophagy, exacerbates chloroquine toxicity in cancer cells [21,22]. A chemical that purely acts to activate autophagy paired with a chemical that purely inhibits autophagy would be predicted to offset the effects of one another, yet, the empirical results are the opposite. Others have found that chloroquine regulates glucose metabolism [70,71]. The interpretation of our current results in the context of previous literature is that HCQ kills cancer cells by concomitant disruption of glycolysis, protein quality control, chromosome condensation and segregation, and cellular trafficking, not solely by inhibition of autophagy.

Biomarker development for HCQ or chloroquine clinical trials may utilize the broad spectrum of data released here. While improvements remain needed, one of the most successful biomarker tests in ovarian cancer does not utilize a lone single gene, but rather assesses a combination of mutations within homologous recombination repair pathway genes as well as genome-wide chromosome alterations and telomere imbalance [72,73]. For HCQ trials, it is now feasible to utilize transcriptome-inclusive diagnostic panels to compare with translational science screen hits [74,75]. Transcriptome diagnostics may consider utilizing transcriptionally upregulated genes within the HCQ-evolved cells (Supplemental Table S2) as negative predictors for responders, while CRISPR-Cas9 sensitizing hits (Supplemental Table S5) would be positive predictors of responders in transcriptionally downregulated genes identified within an individual patient’s tumor. As a simplified single-gene example from our dataset, since NEK2 is upregulated in many cancers [76], we propose this may be one biomarker within a biomarker panel which, when overexpressed, may be predictive of patients who fail HCQ-based therapies.

## List of abbreviations

ER endoplasmic reticulum

FDA food and drug administration

HCQhydroxychloroquine

indel insertion/deletion mutation

NDAnew drug application

NGS next-generation sequencing

scRNA-seq single-cell RNA sequencing

SNV single nucleotide variant

WES whole-exome sequencing

## Supplementary Material

TableS4_scRNAseq_vs_RNAseq_pathways.xlsx

TableS1_HCQevolved_exome_mutations.xlsx

TableS3_RNAseq_SWANnetworkAnalysis.xlsx

no westerns used.pdf

HCQ resistance supplemental figs.pdf

TableS5_CRISPRcas9Screen_HCQ.xlsx

TableS2_RNAseq_HCQevolved.xlsx

TableS6 RNA CRISPR comparison.xlsx

## Data Availability

The datasets used and/or analyzed during the current study are available as supplemental tables, figures, and data. Additional next-generation sequencing raw and processed data are available from SRA (accessions are RNA-seq at PRJNA1023914, exome sequencing at PRJNA1026586, scRNA-seq at PRJNA1027322, and CRISPR-Cas9 at PRJNA1026602) and GEO (accessions are RNA-seq at GSE245133, scRNA-seq at GSE245329, and CRISPR-Cas9 at GSE245215). Code and reference sgRNA data used to count sgRNAs in NGS data are available under https://github.com/jrdelaney/Published_Code/
