# Supplementary material for: Autophagy unrelated transcriptional mechanisms of hydroxychloroquine resistance revealed by integrated multi-omics of evolved cancer cells
Source: Cell Cycle. 2024 Sep 19;23(7-8):796–816. doi: 10.1080/15384101.2024.2402191 (PMC12184169; doi:10.1080/15384101.2024.2402191)
Supplement: no westerns used.pdf [file KCCY_A_2402191_SM0082.pdf]

Data availability - western blots

Western blots were not performed in this study. This page is not applicable.

All genetic raw data is provided in the appropriate main text paragraph, linked to public databases.
