# Supplementary material for: Autophagy unrelated transcriptional mechanisms of hydroxychloroquine resistance revealed by integrated multi-omics of evolved cancer cells
Source: Cell Cycle. 2024 Sep 19;23(7-8):796–816. doi: 10.1080/15384101.2024.2402191 (PMC12184169; doi:10.1080/15384101.2024.2402191)
Supplement: HCQ resistance supplemental figs.pdf [file KCCY_A_2402191_SM0081.pdf]

OVCAR3

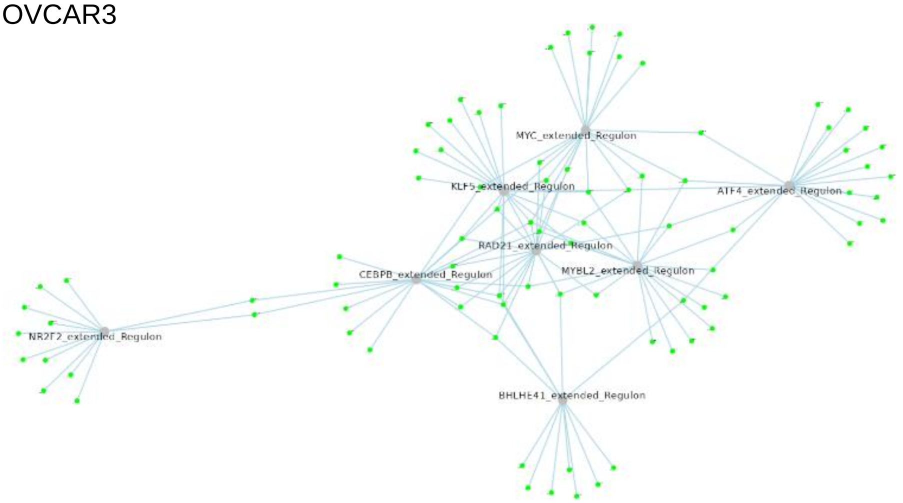

CCL218

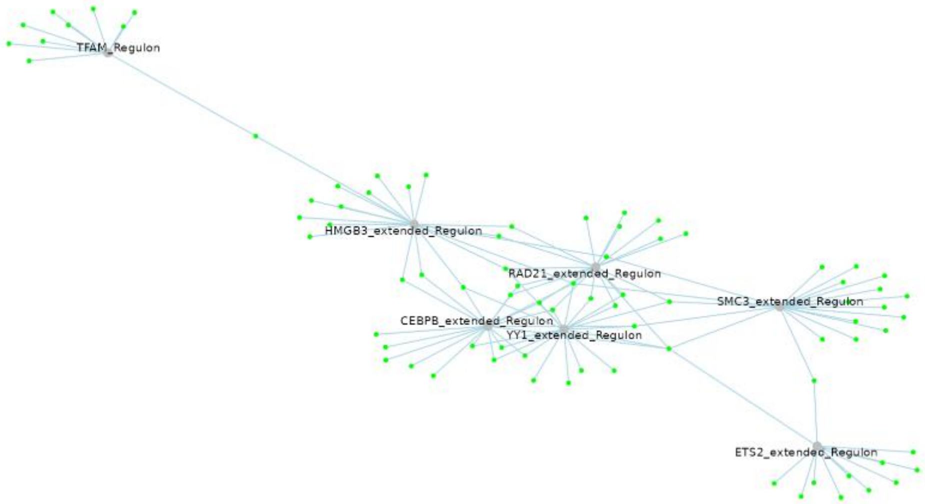

Figure S1. **Single-cell RNA-seq regulons of resistant cells**
SCENIC transcription regulon clustering of scRNA-seq data from the indicated cell lines.

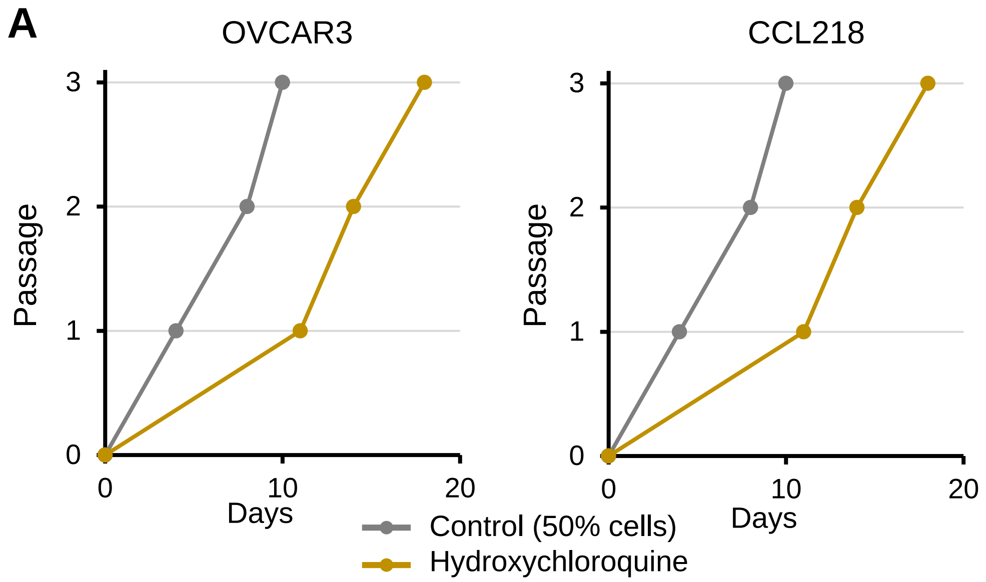

Figure S2. **Quality control of CRISPR-Cas9 screen**
Time points collected for the CRISPR screen are plotted according to what days samples were collected, indicating near confluency was observed. “0” days indicates 4 days after virus administration and 3 days of puromycin selection.
